# TWEAK and TNFα, Both TNF Ligand Family Members and Multiple Sclerosis-Related Cytokines, Induce Distinct Gene Response in Human Brain Microvascular Endothelial Cells

**DOI:** 10.3390/genes13101714

**Published:** 2022-09-24

**Authors:** Delphine Stephan, Anais Roger, Jehanne Aghzadi, Sylvie Carmona, Christophe Picard, Jean-Philippe Dales, Sophie Desplat-Jégo

**Affiliations:** 1 CNRS, INP, Institute of Neurophysiopathol, Aix-Marseille University, 13005 Marseille, France; 2 Aix-Marseille University, CNRS, INSERM, CIML, Centre d’Immunologie de Marseille-Luminy, 13002 Marseille, France; 3 Immunogenetics Laboratory, Etablissement Français du Sang PACA Corse, 13005 Marseille, France; 4 Assistance Publique-Hôpitaux de Marseille, Hôpital de la Timone, BioGénopôle, Service d’Immunologie, 13005 Marseille, France

**Keywords:** TWEAK, TNFα, blood–brain barrier, multiple sclerosis, transcriptomic profiling, brain endothelial cells, leukocyte extravasation, actin cytoskeleton

## Abstract

Tumor necrosis factor-like weak inducer of apoptosis (TWEAK) is a member of the TNF ligand family involved in various diseases including brain inflammatory pathologies such as multiple sclerosis. It has been demonstrated that TWEAK can induce cerebrovascular permeability in an in vitro model of the blood–brain barrier. The molecular mechanisms playing a role in TWEAK versus TNFα signaling on cerebral microvascular endothelial cells are not well defined. Therefore, we aimed to identify gene expression changes in cultures of human brain microvascular endothelial cells (hCMEC/D3) to address changes initiated by TWEAK exposure. Taken together, our studies highlighted that gene involved in leukocyte extravasation, notably claudin-5, were differentially modulated by TWEAK and TNFα. We identified differential gene expression of hCMEC/D3 cells at three timepoints following TWEAK versus TNFα stimulation and also found distinct modulations of several canonical pathways including the actin cytoskeleton, vascular endothelial growth factor (VEGF), Rho family GTPases, and phosphatase and tensin homolog (PTEN) pathways. To our knowledge, this is the first study to interrogate and compare the effects of TWEAK versus TNFα on gene expression in brain microvascular endothelial cells.

## 1. Introduction

Tumor necrosis factor-like weak inducer of apoptosis (TWEAK) is a ligand member of the TNF super family, expressed as both a membrane-bound and soluble form [1]. TWEAK interacts with its receptor Fn14 and triggers the activation of multiple pathways, reviewed by Bhattacharjee et al. [2]. It is now accepted that TWEAK is involved in many human pathologies, including brain inflammatory diseases [3]. Specifically, TWEAK’s role in multiple sclerosis (MS) has been delineated previously: its level in postmortem tissue is increased in MS patients [4], overexpression of TWEAK exacerbates the clinical symptoms of the experimental autoimmune encephalomyelitis (EAE), a murine MS model [5], and anti-TWEAK antibodies injections decrease the phenotype of the same EAE model [6]. Moreover, high levels of serum-soluble TWEAK are associated with neuroinflammation [7]. Our team has also previously shown that TWEAK stimulation of an in vitro blood–brain barrier (BBB) model using human brain endothelial cells (hCMEC/D3) increases the permeability of the barrier by increasing the expression of matrix metalloproteinase-9 (MMP-9) [8]. Furthermore, endothelial cells (ECs) and astrocytes, two of the major cellular components of the BBB, are targets of TWEAK [9,10].

The BBB is a specialized physical and metabolic barrier between the central nervous system (CNS) and its surroundings, specifically serving as a selective border control for molecules and cells from the periphery to the brain. The BBB’s integrity is ensured by the neurovascular unit’s cellular players such as ECs, astrocytes, pericytes, microglia, and neurons as well as components of the extracellular matrix. Increased permeability of the BBB is an early event in the development and progression of various brain inflammatory diseases including MS.

MS is a chronic autoimmune inflammatory disorder of the CNS mediated by immune cells including T cells, B cells, and macrophages that enter a damaged BBB and target the myelin sheath around neurons. Although the specific and intricate etiology of the disease is not well understood, it is well established that the dysregulated and aberrant entry of peripheral immune cells into the brain parenchyma is a key feature of the disease. Extravasating cells can be trafficked into the cerebral space by being attracted to the site by chemokines, attaching to EC receptors and entering the cerebral space through dysfunctional junctions between ECs. Current medication exists to block receptors in order to interrupt entry through the BBB, but targeting chemokines and junction proteins provides an additional avenue for therapeutic solutions.

The mechanisms by which TWEAK regulates and remodels the BBB and therefore contributes to the pathogenesis of MS remain unclear. To gain more insight, we wanted to compare the modulation of ECs by TWEAK and TNFα and to determine any differences these related cytokines exert on the BBB. In this study, we sought to examine the transcriptional profile change of human BBB ECs upon TWEAK versus TNFα exposure.

## 2. Materials and Methods

### 2.1. Cells and Cell Culture Reagents

The human brain microvascular endothelial cell line (hCMEC/D3) was a kind gift from Dr. Pierre-Olivier Couraud’s team (Paris, France) [11]. hCMEC/D3 cells were seeded on Transwell^®^ filters (polycarbonate 6 well-plates, pore size 3.0 μm, Corning, Lowell, MA, USA) coated with type I collagen (BD Biosciences, Paris, France), at a density of 235,000 cells/cm^2^ in commercially available complete medium EGM™️-2MV, supplemented with vascular endothelial growth factor, insulin-like growth factor 1, epidermal growth factor, basic fibroblast growth factor, hydrocortisone, ascorbate, penicillin-streptomycin, and 2.5% fetal calf serum (all from Lonza, Walkersville, MD, USA). For differentiation and expression of junction-related proteins, the hCMEC/D3 cells were grown at confluence in a growth-factor-depleted medium.

Primary human umbilical vein endothelial cells (HUVECs) were obtained from ATCC (Manassas, VA, USA) and were cultured in EGM™️-2 complete medium. The human cervical adenocarcinoma cell line (HeLa) was obtained from ATCC and was maintained in Dulbecco’s Modified Eagle’s Medium (DMEM) supplemented with 10% fetal calf serum and 1% penicillin-streptomycin. All cells were cultured in a 37 °C 5% CO_2_ humidified and 95% O_2_ atmosphere incubator.

For stimulation assays, cells were incubated for 3 h, 12 h, or 24 h with recombinant human TWEAK (100 ng/mL, Peprotech,,Neuilly-sur -Seine, France) or recombinant human TNFα (10 ng/mL, Peprotech, Neuilly-sur -Seine, France ) [8]. All reagents were endotoxin-free.

### 2.2. Microarray Assay

For microarray-based transcriptomic analysis, hCMEC/D3 monolayers, stimulated or not with either TWEAK or TNFα for 3 h, 12 h, or 24 h, were prepared for RNA isolation using the NucleoSpin RNA Plus kit (Macherey-Nagel, Hoerdt, France). The transcriptome analysis was performed on human whole genome oligo microarrays from Agilent company at the TAGC Transcriptomic Platform (Marseille, France; https://tagc.univ-amu.fr, accessed on 20 September 2022). Sample amplification, labeling, and hybridization were performed in line with the Agilent One-Color Microarray-Based Gene Expression Analysis (Low Input Quick Amp Labeling) protocol (Agilent Technologies Palo Alto, CA, USA). Briefly, total RNA was reverse transcribed into complementary DNA (cDNA) using the T7 promoter primer. Synthesis of cyanine-3-labeled complementary RNA (cRNA) from cDNA was performed in a solution containing dNTP mix, T7 RNA polymerase, and cyanine 3-dCTP and then incubated at 40 °C for 2 h. Labeled cRNA was purified and fragmented before hybridization on Agilent Human Gene Expression 4X44K Arrays (Agilent Technologies, Palo Alto, CA, USA) at 65 °C for 17 h. Raw microarray signals were scanned and extracted using Agilent Feature Extraction Software (Agilent Technologies, Santa Clara, CA, USA). Quality control and data normalization using quantile methods and a background correction were applied on a BioAnalyzer Agilent 2100 (Agilent Technologies, Palo Alto, CA, USA). Raw signals with a fluorescence intensity of less than 100 were excluded for further analysis. For the differential gene expression analysis on microarrays, the values of the induction and inhibition of TWEAK or TNFα cytokines versus control (fold change (FC)) at the different timepoints were selected based on a FC > 1.5 or < −1.5, respectively. In case of discordance between FC duplicates, the pointed gene was excluded from the analysis. We analyzed genes that displayed a significant increase or decrease. Consequently, for the biological interpretation, 16,389 genes for TNFα and 16,763 genes for TWEAK were included from the 3 h time point; 14,455 genes for TNFα and 12,577 genes for TWEAK were included from the 12 h time point; 13,017 genes for TNFα and 14,469 genes for TWEAK were included from the 24 h time point.

Biological interpretation of the transcriptomic data was performed using Qiagen Ingenuity Pathway Analyses (IPA) following instructions provided by Qiagen (Ingenuity Systems Inc., Redwood City, CA, USA; www.ingenuity.com, accessed on 20 September 2022). Similarities and differences in canonical pathways were analyzed. The rationale of using IPA is that it utilizes expertly curated biological interactions and functional annotations from multiple databases. The significance of the association between the data set and the canonical pathway was determined based on two parameters: (i) a ratio of the number of genes from the data set that map to the pathway divided by the total number of genes that map to the canonical pathway and (ii) a *p*-value calculated using Fischer’s exact test determining the probability that the association between the genes in the data set and the canonical pathway is not due to chance alone. The Z-score algorithm was designed to reduce the chance that random data will generate significant predictions. IPA predicts that the canonical pathway is significantly increased when Z-score ≥ 2 and is significantly decreased when Z-score ≤ −2. Results were considered statistically significant when *p* ≤  0.05 (*), *p*  ≤  0.01 (**), or *p*  ≤  0.001 (***). Microarray raw data are available in the ArrayExpress database under accession number E-MTAB-12222.

### 2.3. Real-Time Quantitative PCR (qPCR)

Total RNA was isolated from cultures of hCMEC/D3 using the NucleoSpin RNA Plus kit (Macherey-Nagel, Hoerdt, France). Single-strand cDNA was synthesized from 500 ng of total RNA using oligo(dT)_12–18_ primers (Invitrogen, Thermo Fisher Scientific, Villebon-sur-Yvette, France) and Moloney murine leukemia virus reverse transcriptase (Invitrogen), according to the manufacturer’s instructions. qPCR experiments were carried out with the 7500 Fast Real-Time PCR System (Applied Biosystems, Life Technologies SAS, Courtaboeuf, France). All reactions were performed using TaqMan Fast Universal PCR Master Mix (Applied Biosystems, Life Technologies SAS, Courtaboeuf, France ) and different probes from the TaqMan Gene Expression Assays with the following references: CLDN5, Hs00533949_s1; JAM1, Hs00375889_m1; THY1, Hs00174816_m1; CXCL12, Hs03676656_mH, according to the manufacturer’s instructions (Applied Biosystems, Life Technologies SAS, Courtaboeuf, France). The Abelson gene was used as reference with the laboratory designed oligo ABL-F (5′-TGGAGATAACACTCTAAGCATAACTAAAGGT-3′), ABL-R (5′GATGTAGTTGCTTGGGACCCA-3′) and ABL-TaqMan reverse probe (5′Fam6CCATTTTTGGTTTGGGCTTCACACCATT-Tamra-3′). Each experiment used 12.5 ng of previously prepared hCMEC/D3 cDNA. Samples were run in duplicates on the same 96-well plates and analyzed with the 7500 Software v2.0 (Applied Biosystems, Life Technologies SAS, Courtaboeuf, France). The thermal cycling conditions started with initial denaturation at 95 °C for 20 s, followed by 40 cycles of denaturation at 95 °C for 3 s and annealing and extension at 60 °C for 30 s. Relative expression levels were determined according to the ΔΔCt method where the expression level of the mRNA of interest is given by 2^−ΔΔCT^ where ΔΔCT = ΔCT target mRNA − ΔCT reference mRNA (Abelson) in the same sample.

### 2.4. Western Blot Analysis

hCMEC/D3 that were treated for 24 h with TWEAK or left untreated and control cells HUVEC and HeLa were lysed in RIPA buffer supplemented with phosphatase and protease inhibitor cocktail (Roche Applied Science, Mannheim, Germany). Lysates were sonicated and protein concentrations were determined using the Lowry method (Bio-Rad, Hercules, CA). After boiling, aliquots containing 30 µg total protein were loaded in Laemmli Buffer and separated by 15% SDS PAGE using a MiniBlot system (Bio-Rad). Proteins were transferred onto nitrocellulose membranes (Amersham Biosciences, Buckinghamshire, UK) in transfer buffer (25 mM Tris, 192 mM glycine, and 20% ethanol). Membranes were blocked in TBS, 3% BSA (Sigma-Aldrich St. Louis, MO, USA), 0.1% Tween 20 and then probed with the primary antibody against claudin-5 (1 µg/mL) (Neo Biotech, Nanterre, France) or β-actin (0.2 µg/mL) (Sigma-Aldrich St. Louis, MO, USA), diluted in blocking buffer overnight at 4 °C. After washing, membranes were incubated with a peroxidase-conjugated secondary antibody (Thermo Fisher Scientific, Waltham, MA, USA). Finally, proteins were detected using a chemiluminescence kit (Amersham Biosciences, Buckinghamshire, UK). Membranes were digitized using Nine-Alliance imaging system (UVITEC Cambridge, UK), and optical densities of the bands were quantified using Image J densitometric software (https://imagej.nih.gov/ij, accessed on 20 September 2022). The experiment was performed in triplicate for each condition.

## 3. Results

### 3.1. Modulation of Genes Involved in Leukocyte Extravasation at the 24 h Timepoint

Due to TWEAK’s suggested role in orchestrating leukocyte extravasation, we first ran a supervised transcriptomic analysis on hCMEC/D3 cells incubated with TWEAK versus TNFα at the 24 h timepoint and analyzed their effects on specific genes belonging to this signaling pathway. To understand what part of the cascade the cytokines modulated, differentially expressed gene (DEG) ratios (treated versus control hCMEC/D3 cells) were filtered based on their FC. DEGs belonging to leukocyte extravasation signaling after TWEAK or TNFα treatment after 24 h as determined by IPA are illustrated in Table 1. As can be seen, the list includes adhesion molecules (THY1, ITGA3, PECAM1, CD44), a chemokine (CXCL12), enzymes (PIK3C2B, PRKCZ, MMP9), and tight junction (TJ) proteins (CLDN11, CLDN5, JAM1, CLDN23).

The most significant increase in mRNA involved in leukocyte extravasation mediated by TWEAK was for GNAI2 and the most significant decrease was seen with PRKCZ. In both these cases, TNFα generated no significant variation on gene expression. In addition, of the 18 genes we looked at, 5 had the same directionality when stimulated with TWEAK or TNFα (MMP9, CCXCL12, CLDN5, JAM1, PIK3C2B) but only CXCL12 was upregulated by both (FC > 1.5).

To compare the results of the microarray studies to a second established transcriptomic method, qPCR was performed to assess the mRNA expression levels of 4 selected genes (*CLDN5, JAM1, THY1, CXCL12*) implicated in leukocyte extravasation in Table 1. Comparison of FC values between microarray and qPCR methods are shown in Table 2. CLDN5 showed the most robust correlation between results obtained by microarray and qPCR, displaying a significant decrease in expression after either TWEAK or TNFα exposure. THY1, CXCL12, and JAM1 showed inconsistent directionality of results between qPCR and microarray data for either TWEAK or TNFα exposure.

### 3.2. Claudin-5 Protein Expression after 24 h TWEAK Stimulation of hCMEC/D3 Cells

**Figure 1:**
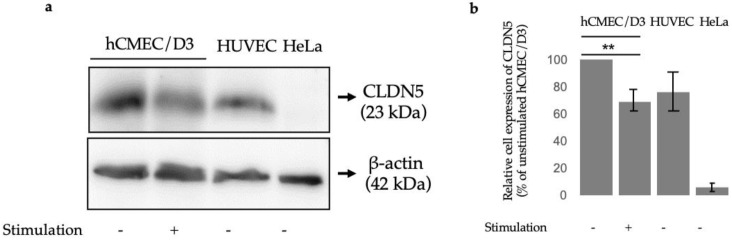
Claudin 5 protein expression levels (**a**) Representative immunoblots showing protein levels of claudin 5 (CLDN5) in hCMEC/D3 cells after no stimulation (-) or after TWEAK (100 ng/mL) stimulation (+) as compared to control cells (HUVEC and HeLa). (**b**) Quantification of Western blot normalized to unstimulated hCMEC/D3 cells; *p* ≤  0.01 (**).

At the protein level, we have chosen to illustrate claudin-5 expression variation as it is a characterizing TJ protein of BBB ECs [12] and is downregulated when exposed to MS sera [13]. At the mRNA level, we found that TWEAK decreases CLDN5 expression at all three time points, but TNFα exerts no effects on CLDN5 mRNA at 24 h (Table 1) and even at 3 h or 12 h (Supplementary Table S1). We measured CLDN5 protein expression in untreated or 24 h-TWEAK-treated hCMEC/D3 cells and also in unstimulated HUVEC and HeLa cells using Western blotting. As expected, HUVECs expressed some claudin-5 and HeLa did not [14,15], however, we observed that claudin-5 expression was significantly downregulated in hCMEC/D3 cells treated with TWEAK for 24 h compared to control conditions (Figure 1).

### 3.3. Overall Transcriptional Profiles of Human Brain Microvascular Endothelial Cells in Response to TWEAK or TNFα at 3, 12, and 24 h

**Figure 2:**
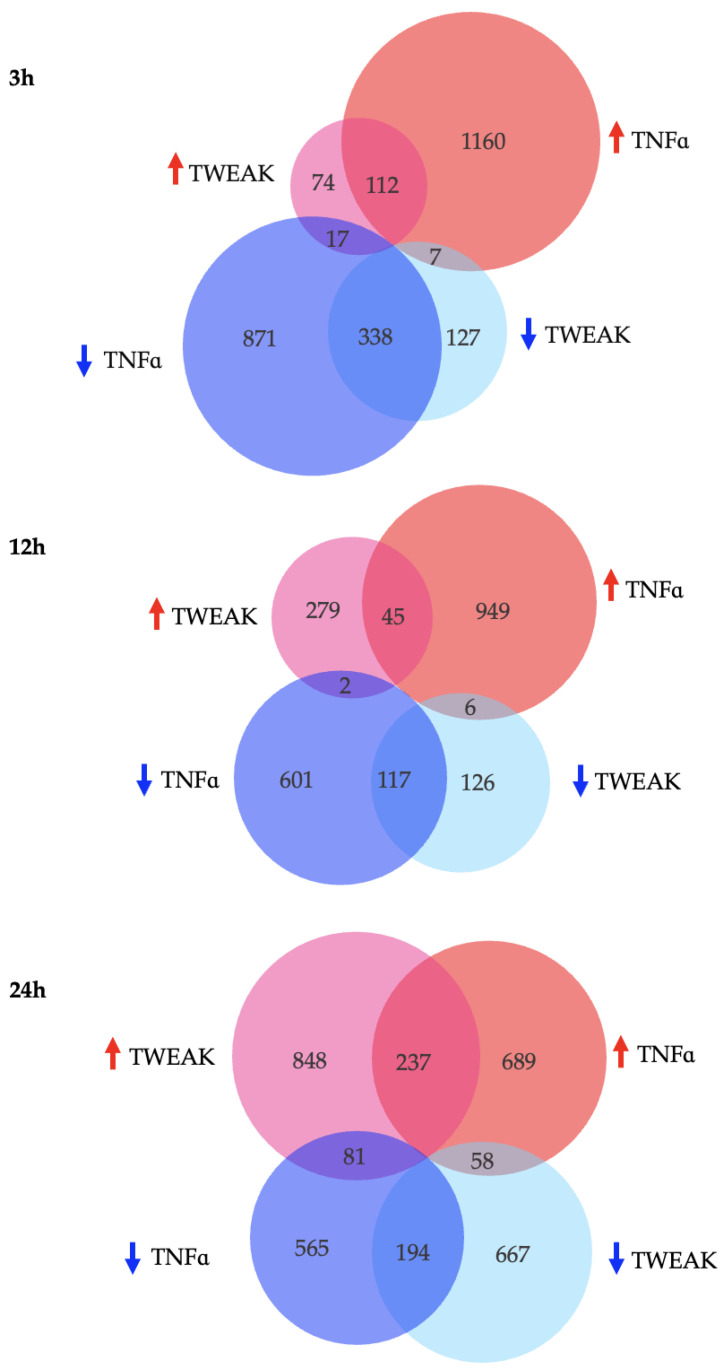
Number, distribution and directionality of shared and unique DEGs for hCMEC/D3 cells treated with TWEAK or TNFα after 3 h, 12 h, and 24 h. Pink (TWEAK) and Red (TNFα) circles: upregulated genes (FC > 1.5), turquoise (TWEAK) and blue (TNFα) circles: downregulated genes (FC < −1.5).

We next sought to compare the overall transcriptional profiles of cells stimulated by TWEAK or TNFα and how their modulation of genes overlapped. We catalogued the number of genes whose levels were significantly altered after 3 h, 12 h, and 24 h of TWEAK or TNFα exposure (Figure 2). While TNFα stimulation strongly modulated the number of DEGs after 3 h, TWEAK only reached a similar magnitude of DEGs after 24 h. After 24 h, the number of genes affected were globally similar between the two cytokines, with TNFα slowly decreasing the amount of DEGs between 3 h and 24 h, and TWEAK dramatically increasing the number of DEGs only between the two last time points.

In order to evaluate unique and common deregulated genes in hCMEC/D3 cells after stimulation with TWEAK and TNFα, we also compared their common DEG profiles (Figure 2). We noted a decrease in overlap of DEGs during the middle time point, with approximately 8% of genes in common at 12 h as opposed to 18% and 17% after 3 h and 24 h stimulation, respectively (percentages correspond to the ratio: overlapping genes to overlapping genes and non-overlapping genes). Of the overlapping genes, 95% of them were modulated in the same way after 3 h and 12 h, and this value decreased to 76% at 24 h (percentages correspond to the ratio: overlapping genes that are modulated in the same direction to total overlapping genes).

### 3.4. Canonical Pathway Analysis after 24 h TWEAK or TNFα Incubation

To gain insights into the effects of TWEAK versus TNFα on overall cellular pathways, we used IPA. The top pathways are listed in Table 3 according to their levels of activation (expressed as Z-scores) if their *p*-value < 1 × 10^−2^ after 24 h of TWEAK treatment. Significantly activated canonical pathways by TWEAK exposure predicted by IPA included integrin signaling, VEGF signaling, actin cytoskeleton signaling, ephrin receptor signaling, signaling by Rho family GTPases, regulation of actin-base motility by Rho, amyotrophic lateral sclerosis signaling, IL-8 signaling, RhoA signaling, Rac signaling, and NGF signaling. Among these, only integrin signaling, actin cytoskeleton signaling, ephrin receptor signaling, and RhoA signaling were also significantly upregulated by TNFα. Neuroinflammation signaling pathway and leukocyte extravasation signaling showed an increase after TWEAK stimulation, albeit not a significant one (Z-score > 1.5), and were almost unaffected by TNFα. PTEN and RhoGDI signaling pathways were significantly inhibited (Z-score < −2) by TWEAK exposure, and of these only PTEN was significantly downregulated by TNFα. Two pathways that were significantly increased by TNFα exposure but had virtually no difference during TWEAK incubation were the death receptor signaling and the sirtuin signaling pathway.

## 4. Discussion

In this study, we investigated the transcriptomic profile of a human brain microvascular endothelial cell line (hCMEC/D3) after exposure to two MS-related pro-inflammatory cytokines TWEAK and TNFα. We compared the gene profiles of these cells after 3 h, 12 h, and 24 h of stimulation and indexed the signaling pathways modulated. We found that, although historically considered as a weaker version of TNFα, TWEAK influenced mRNA expression differently than TNFα. In particular, our goal was to elucidate how TWEAK influences the architecture of the BBB, as this has been of interest in the context of MS pathophysiology.

We first wanted to probe genes involved in leukocyte extravasation to further our understanding of how TWEAK mediates immune cells infiltration across the BBB during MS. As expected, we saw a significant down-regulation of claudin-5, particularly through qPCR data. Claudin-5 is the most ubiquitously expressed TJ protein in mouse brain capillary endothelial cells [12]. The most significant increase in mRNA involved in leukocyte extravasation mediated by TWEAK was for GNAI2, which codes for Gαi2, the G-protein subunit α i2. Luissint et al. have previously shown that this protein immunoprecipitates with claudin-5, and that siRNA-mediated depletion of Gαi2 or claudin-5 led to an increased loosening of the BBB [16]. It was interesting to see here that their mRNA levels were inversely related, maybe as a compensatory mechanism to regain barrier integrity due to a decrease of CLDN5 expression. GNAI2 levels at 3 h and 12 h stimulation were not significantly changed by TWEAK incubation, while CLDN5 displayed persistent decrease at all timepoints (Supplementary Table S1). The same work found that p120-catenin, encoded by CTNND1, also associated with claudin-5 [16]. Our results indicate that CTNND1 followed the same pattern as Gαi2, where it increased after TWEAK exposure as CLDN5 decreased. Furthermore, as expected and observed in a previous study our team led at the protein level, we noted an increase in MMP-9 at the mRNA level [8]. We also saw a significant TNFα-induced increase in expression of PECAM-1, which is involved in recruitment of neutrophils into inflammatory sites. This finding is contrary to what Stewart et al. had previously observed [17]. TWEAK did not significantly modify this gene’s expression levels here. With regards to another adhesion molecule, we have previously reported that following 24 h TWEAK stimulation, hCMEC/D3 cells showed an upregulation in ICAM-1 expression by flow cytometry [8]. In contrast, the present study found that neither TWEAK nor TNFα modulate the expression of ICAM-1 mRNA at any time point significantly (Supplementary Table S1). Thus, all together, this shows that maybe TWEAK rather selectively increases the direct mechanistic breakdown of the BBB (via the decrease of CLDN5 and increase of MMP-9), thus permeabilizing it for easier leukocyte entry, or that perhaps TWEAK participates in recruiting lymphocytes rather than neutrophils by directly influencing ICAM-1′s protein expression. This is further consolidated by the observation that TWEAK did not cause differential expression of CD44 mRNA, another putative adhesion receptor (Table 1). The decrease in PRKCZ expression also fits into the narrative of TWEAK’s involvement in BBB permeability, as it has been involved in TJ protein disruption [18]. PIK3C2B, an upstream activator of PRKCZ, decreased its mRNA levels when our in vitro model was exposed to TWEAK as well [19].

qPCR was used to validate microarray analysis for a set of leukocyte extravasation genes. Although some of our data were in disagreement, this can be expected [20]. We postulate that the location of the PCR primers and microarray probes could have decreased the correlation of the results, an observation that was previously found when comparing data using these methods [21].

Due to its consistent decrease across all quantification methods as well as its crucial role in BBB integrity, we chose to look at the protein levels of claudin-5 in hCMEC/D3 cells after TWEAK treatment. Mirroring its mRNA levels, claudin-5 protein expression was significantly reduced by TWEAK incubation (Figure 1). One of the key features of MS is an opening of the BBB resulting in an influx of peripheral immune cells into the brain parenchyma, in part due to a reduction of TJ proteins [22]. Since the most abundantly expressed TJ protein in BBB ECs is claudin-5 by a 600-fold higher mRNA level compared to other claudin family members, it is interesting to see that TWEAK modulates its protein levels as well, which may reveal one of TWEAK’s roles in MS etiology [12]. Previous studies utilizing knockout and knockdown mouse models have shown that the lack of claudin-5 increased permeation of tracer molecules and even resulted in the death of mice within 10 h of their birth [22,23]. Importantly, the breakdown of the BBB and the loss of TJ proteins including claudin-5 was confirmed in postmortem studies on human brains [24]. This indicates that TWEAK is a relevant player in the permeabilization of the BBB, which is itself a precursor to inflammatory cells influx into the cerebral space [25]. Blecharz et al. showed that when cEND cells, a murine brain endothelial cell line, were incubated with sera from MS patients, claudin-5 decreased and MMP-9 increased, consistent with our observations in this study [13]. Additionally, the relationship between TWEAK and TJ protein dysregulation reaches to further neurological diseases where the BBB’s architecture is disturbed, for instance in schizophrenia [26]. Two recent studies found that TWEAK serum levels are significantly elevated in schizophrenic patients [27], and claudin-5 levels are significantly lowered [28]. Thus, restoring claudin-5 function via TWEAK inhibition could serve as a potential therapeutic solution for multiple neurodegenerative diseases, and MS in particular. Future studies could look at the effects of administrating anti-TWEAK antibodies like our team has previously done [6] and delineate its effects on claudin-5 activity and subsequent BBB integrity.

TWEAK was long considered a less strong “cousin” of TNFα that contributed similarly to different inflammatory diseases, including the EAE model of MS [5,6,29]. However, within a few years of its original discovery, TWEAK proved to be a multifaceted cytokine on cellular responses that did not always match how its family member TNFα behaved. Studies quickly noticed that TWEAK could in fact modulate and balance TNFα activity by opposing its effects [30]. Given this curious relationship, we interrogated their effects upon stimulated human brain microvascular endothelial cells. Overall, TNFα led to more DEGs compared to TWEAK during the first hours of stimulation, but after 24 h, this difference was minimal, and TWEAK was modulating virtually the same number of genes as its family member (Figure 2). This indicates that chronic exposure to TWEAK, whose levels are increased in MS patient sera [7], might start behaving like a stronger pro-inflammatory cytokine.

Finally, we were interested in comparing how TWEAK and TNFα incubation affected certain canonical pathways. We were initially interested in evaluating the cytokines’ effects on leukocyte extravasation, as these effects were seen by our group and others in previous works [8,31,32]. We found that TWEAK had a significant effect on 13 signaling pathways, all in which its effects were stronger than those engendered by TNFα after 24 h. We have provided a summary of these findings in Table 3 to serve as a point of reference for further exploration.

Our results suggest that increased permeability in stimulated brain microvascular endothelial cells by TWEAK could be mediated by Rho GTPases-dependent cytoskeleton remodeling. TNFα has been shown to induce endothelial cells activation by actin cytoskeletal rearrangement, with an increase in actin stress fiber formation followed by intercellular gap formation [33,34]. Similarly, actin cytoskeleton signaling was shown to be significantly activated by TNFα as well by TWEAK in our present study. Well-known regulators of VE-cadherin, one of the most essential adhesion molecules controlling endothelial integrity [35], and the actin cytoskeleton are the Rho family GTPases. Of this family, Rac1 and Cdc42 promote endothelial barrier function, whereas RhoA induces contraction-driven endothelial hyperpermeability [34]. RhoA is extensively described as a key regulator of vascular leakage [36] and leukocyte trans-endothelial migration [37]. Rho GTPases are inactivated by Rho GDIs. It has been previously established that TNFα activates RhoA and Rac signaling [38], as found in our present work, but it did not affect RhoGDI signaling. We have found, here, that RhoA and Rac signaling were activated whereas RhoGDI signaling was inhibited by TWEAK. Zhang et al. also showed that there is an accumulation of RhoA^+^ cells in lesions in EAE mice, indicating that it has an important role in the effector phase of MS and that its inhibition might be therapeutic for MS patients [39]. Therefore, RhoA’s modulation through TWEAK inhibition could also provide a treatment option to be considered.

Stimulation of brain microvascular ECs by TWEAK enhanced VEGF and suppressed PTEN signaling. On the other hand, TNFα suppressed PTEN signaling but did not significantly increase VEGF signaling. In VEGF-stimulated ECs, an increase in endothelial permeability was observed [40]. In brain microvascular endothelial cells, a downregulation of CLDN5 was concomitant with VEGF action and correlated with disruption of the BBB [41]. PTEN is known to modulate VEGF signaling and function in endothelial cells [42,43]. Furthermore, a recent report showed that deletion of Pten in cerebrovascular ECs led to an increased transcellular permeability of the BBB [44]. Thus, TWEAK’s downregulation of the PTEN axis and enhancement of the VEGF pathway could also explain pervasive effects of this cytokine on hallmark MS features, notably the BBB’s disintegration.

We observed that, contrary to TNFα, TWEAK had no effect on the death receptor signaling, which contradicts previous results that delineated its involvement in this pathway [45]. We also found that BAX was significantly upregulated by TWEAK (FC = 3.95) and downregulated by TNFα (FC = −2.00) (Supplementary Table S1). This suggests that TWEAK might induce apoptosis through a different mechanism than its family member rather than by following a similar pathway as previously reported [46].

To our knowledge, this is the first study to systematically profile the effects of TWEAK versus TNFα on the transcriptomics of human brain microvascular endothelial cells. Our data provide new exploratory routes to consider when investigating BBB permeability as an initial mechanism in the physiopathology of MS. Given its role in the breakdown of the barrier, TWEAK is also a potential candidate in therapeutic approaches for MS and other disorders. Further combined genomic and proteomic studies are necessary to find out more details on the hierarchy and regulation of DNA-RNA-protein dynamics.

## Figures and Tables

**Table 1:** Differential expression of IPA-identified genes belonging to leukocyte extravasation signaling in hCMEC/D3 cells after 24 h of TWEAK or TNFα treatment. Red: upregulated genes (FC > 1.5), blue: downregulated genes (FC < −1.5). FCs with values between −1 and 1 were not included in IPA-run analysis.

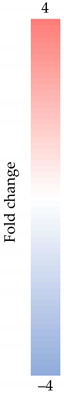	**Gene**	**TWEAK**	**TNFα**
	GNAI2	2.913	
	PXN	2.824	−1.453
	ACTC1	2.447	
	THY1	2.400	−1.650
	RAC2	2.217	−1.045
	CTNND1	2.199	
	ACTB	1.988	−1.421
	MMP9	1.912	1.206
	ITGA3	1.658	
	CLDN11	1.646	−1.329
	CXCL12	1.571	3.635
	PECAM1		2.791
	CD44		−2.058
	CLDN5	−1.771	−1.153
	JAM1	−1.988	−1.332
	CLDN23	−2.214	1.952
	PIK3C2B	−2.549	−1.093
	PRKCZ	−2.889	

**Table 2:** Comparison of FCs of DEGs belonging to leukocyte extravasation signaling obtained via microarray and qPCR assays performed on hCMEC/D3 cells after 24 h incubation in TWEAK or TNFα. Upregulated genes (FC > 1.5) are in red and downregulated genes (FC < −1.5) are in blue.

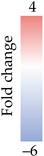	**Gene**	**TWEAK**		**TNFα**	
		**Microarray**	**qPCR**	**Microarray**	**qPCR**
	THY1	2.40	−1.11	−1.65	1.14
	CXCL12	1.57	−1.04	3.64	2.17
	CLDN5	−1.77	−6.08	−1.15	−4.99
	JAM1	−1.99	−1.47	−1.33	1.11

**Table 3:** Top canonical pathways significantly modulated by 24 h of TWEAK treatment compared to TNFα. Z-score and significance of *p*-value after 24 h stimulation with TWEAK and TNFα for comparison. Sirtuin signaling pathway and death receptor signaling are included due to their statistical significance after TNFα treatment. Red and blue represent up- and down-regulated pathways, respectively. Results were considered statistically significant when *p*  ≤  0.05 (*), *p*  ≤  0.01 (**), or *p*  ≤  0.001 (***).

	TWEAK		TNFα	
	Z-Score	*p*-Value	Z-Score	*p*-Value
Canonical Pathways				
Integrin Signaling	3.570	***	2.449	**
VEGF Signaling	3.530	***	1.698	***
Actin Cytoskeleton Signaling	3.430	***	2.335	***
Ephrin Receptor Signaling	3.380	***	2.887	
Signaling By Rho Family GTPases	3.240	***	1.134	***
Regulation Of Actin-base Motility By Rho	2.887	**	1.732	**
Amyotrophic Lateral Sclerosis Signaling	2.830	***	0.577	
IL-8 Signaling	2.530	***	1.800	***
RhoA Signaling	2.357	**	2.183	**
Rac Signaling	2.183	**	1.414	***
NGF Signaling	2.065	**	0.302	
Neuroinflammation Signaling Pathway	1.915	*	0.174	**
Leukocyte Extravasation Signaling	1.877	**	−0.218	**
Death Receptor Signaling	0.000	**	2.138	**
Sirtuin Signaling Pathway	−0.392	**	2.400	**
RhoGDI Signaling	−2.236	**	−0.218	**
PTEN Signaling	−3.578	***	−2.065	***

## Data Availability

Data analyzed during this study include an additional file available on the website of Genes, “Supplementary Table S1”.

## Supplementary Materials

The following supporting information can be downloaded at: https://www.mdpi.com/article/10.3390/genes13101714/s1, Table S1: Microarray processed data
